# Case Report: A novel intronic variant of *NIPBL* gene detected in a child with cornelia de lange syndrome

**DOI:** 10.3389/fgene.2025.1665167

**Published:** 2025-09-10

**Authors:** Xiao Ting Shao, Yu Xuan Dai, Yu Fang Zhao, Yu Hang Chen, Ling Jing Ying

**Affiliations:** Department of Pediatrics, Jinhua Municipal Central Hospital, Jinhua, China

**Keywords:** cornelia de lange syndrome, variant, NIPBL, splicing, minigene

## Abstract

**Background:**

Cornelia de Lange syndrome (CdLS) is a genetically heterogeneous disorder involving multi-system organs, causing physical and mental congenital malformation. Nipped-B-like protein (*NIPBL*) variants are associated with various CdLS phenotypes. Newborns with typical clinical manifestations (intellectual disability, special appearances, and limb malformation) require a diagnosis. However, diagnosing CdLS is challenging on account of its heterogeneity of genotype and phenotype.

**Methods:**

In this study, molecular analysis was applied, containing whole exome sequencing (WES), reverse transcriptase PCR (RT-PCR), and minigene splicing assays.

**Results:**

We identified a novel splice-donor variant (*NIPBL* c.6343 + 1G>A) by WES. RT-PCR and minigene splicing assays were performed to identify the function of the splice-donor variant on subsequent RNA splicing. The variant caused exon 36 to be skipped. A premature termination codon (PTC) appeared subsequently and a truncated protein with a length of 2088 aa was produced.

**Conclusion:**

A novel pathogenic variant of CdLS is identified, which affects normal mRNA splicing of the *NIPBL* gene. These findings enrich the knowledge of CdLS gene variants, which may be responsible for developing this rare disease.

## 1 Introduction

Cornelia de Lange syndrome (CdLS), a rare congenital disease of the multisystem caused by genetic variants, has an extremely low incidence of one estimated CdLS per 10,000 to 30,000 live births (Mannini et al., 2013). Children with CdLS have typical clinical manifestations such as facial features, hypertrichosis, growth restriction, intellectual disability, and upper limb deformity (Kline et al., 2018). Occipital skin thickening was found in nearly 51% of patients, with special appearance features accounting for nearly half of (Clark et al., 2012). Approximately 30% of the patients were reported to have an upper limb deficit (Bağış et al., 2020). Although cardiac abnormalities are not the primary standard and typical clinical manifestation of the disease, approximately 50% of patients with CdLS present with congenital heart defects (Ayerza et al., 2017; Dempsey et al., 2014). An international consensus statement created a scoring system defining CdLS as both classical and non-classical CdLS (Kline et al., 2018). Classical CdLS, due to unique facial features, growth limitations, and limb deformities, can be identified at birth by experienced pediatricians or clinical geneticists. However, due to the heterogeneous genotype and phenotype of CdLS, some non-classical CdLS do not have characteristic clinical manifestations, thus bringing great difficulties for clinical diagnosis.

As currently reported in the literature, CdLS is due to variants in the following genes, *NIPBL, SMC1A, SMC3, RAD21, HDAC8, BRD4, and ANKRD11* (Deschamps, 2022). *SMC1A* variants are found in about 5% of patients with CdLS and the variants in *HDAC8*, *RAD21*, and *SMC3* totaled about 5%; *BRD4* and *ANKRD11* gene variants are newly discovered in recent years, being too small to count the percentage; The clinical manifestations of several variants are less typical (Kline et al., 2018). Variants in the *NIPBL* gene are detected in 60% of patients with CdLS (Nizon et al., 2016; Krantz et al., 2004). The *NIPBL* gene is located on 5p13.2 and encodes for a triangular protein that plays an important role in the chromatid cohesion process and enhancer-promoter communication (Nizon et al., 2016; Krantz et al., 2004). Potential pathogenic variants in the *NIPBL* gene have been tightly associated with the typical manifestation of CdLS (Moss et al., 2017; Huisman et al., 2017).

This study reports a classic case of CdLS diagnosed from birth in a child who also had a variant in the *NIPBL* gene, but we detected a novel intronic splice-donor variant.

## 2 Materials and methods

### 2.1 Patient and clinical assessment

The study protocol was approved by the Ethics Committee of Jinhua Central Hospital of Zhejiang Province (approval number No. 2024-43). The study was conducted by the principles of the Declaration of Helsinki. Informed written consent from the proband’s parents was obtained for the publication of this case.

We collected detailed clinical data, including age, sex, birth history (birth size), and characteristic manifestations (including facial features and physical examination). In addition, we collected clinical diagnostic examination results, including ultrasound examination (kidney, testis, hepatobiliary system, cardiac ultrasound examination) and brain magnetic resonance imaging (MRI), and the treatment of the child (medication status and surgery status). Follow-up until 3 years and 5 months of age, the height and weight data were collected. In addition, we also applied a Gesell Developmental Scale to test the patient’s behavior developmental quotient.

### 2.2 Whole exome sequencing

Qualified whole blood samples from cases of the proband and his parents were collected to extract DNA. Method of Aligent SureSelect was used for DNA sample preparations. Agilent SureSelect XT Human All Exon V6 Kit (Agilent, Santa Clara, CA, United States) was used for targeted region capture and gene library preparation, under the guidance of operating instruction. High-throughput sequencing was fulfilled using an Illumina NovaSeq 6,000 system (Illumina, San Diego, CA, United States), the amount of paired-end reads was 150 bp.

### 2.3 Prediction of pathogenicity and splicing effects of the variant

The c.6343 + 1G>A variant is on-site +1 of intron36 (G mutated into A) and belongs to a typical site that affected mRNA splicing in all probability. The Human Splicing Finder (HSF, https://hsf.genomnis.com/login), SpliceAI (https://spliceailookup.broadinstitute.org/), and RDDCSC (https://rddc.tsinghua-gd.org/search-middle?to=SplitToolModel) algorithms were used to determine the effects of the splice-donor variant on the mRNA splicing process of *NIPBL*. Moreover, MutationTaster (https://www.mutationtaster.org/) was used for evaluating the pathogenicity of the splice-donor variant on protein expression.

### 2.4 Reverse transcription polymerase chain reaction and sanger sequencing

Reverse transcription polymerase chain reaction (RT-PCR) was performed to verify the potential influence on mRNA splicing of the c.6343 + 1G>A variant. The whole blood samples of this proband (variant) and his healthy mother (wild type) were used for extracting total RNA, according to a Total RNA Extraction Kit (TR121-50; GenStone, Littleton, CO, United States). The genomes were digested and synthesis of cDNA was performed by reverse transcription based on HifairTM first Strand cDNA Synthesis SuperMix kit (11123ES70; Yeasen, Shanghai, China). The procedure of this reverse transcription was shown as ‘37 °C for 15 min, then at 85 °C for 5 s’. Primers were designed, and the RT-PCR products were amplified through PCR on the condition of ‘95 °C for 30 s, 60 °C for 30 s, 72 °C for 30 s (35 cycles), then 72 °C for 5 min’. Amplified PCR products were tested using the method of 2% agarose gel electrophoresis, then the electrophoretic bands of PCR products were recovered through gel extraction. Sanger sequencing was then performed.

### 2.5 Minigene splicing assay

Minigene splicing experiments (*in vitro* assays) were used to further confirm the function of the variant in *NIPBL* gene splicing. PCR amplification using designed primers was used to obtain variant and wild-type target gene segments with restriction enzyme cutting sites. Four recombinants were acquired by inserting the obtained target minigene into pcDNA3.1 and pcMINI-N vectors. *NIPBL*-pcDNA3.1-wt/mut recombinants was successfully constructed by inserting Exon35 (141bp)-Intron35 (148bp)-Exon36 (94bp)-Intron36 (713bp)-Exon37 (155bp) into pcDNA3.1 plasmid. *NIPBL*-pcMINI-N-wt/-mut recombinants were constructed by inserting Exon35 (141bp)-Intron35 (148bp)-Exon36 (94bp)-partial Intron36 (517bp) into pcMINI-N plasmid. These four recombinants were transfected into HeLa and 293T cells (Rapid Plasmid Mini Kit, 1005250; SIMGEN, Schönefeld, Germany), and the cells were cultured for 48 h. A total of eight RNA extractions were obtained from these cell samples by the application of TRIzol^®^ RNAiso PLUS (9,109; Takara). The Hair first Strand cDNA Synthesis SuperMix for qPCR kit (gDNA digester plus) was used for reverse transcription (11123ES70; Yeasen). PCR amplification was visualized using a method of agarose gel electrophoresis, finally, the product was verified through Sanger sequencing analysis.

## 3 Results

### 3.1 Case presentation

**FIGURE 1:**
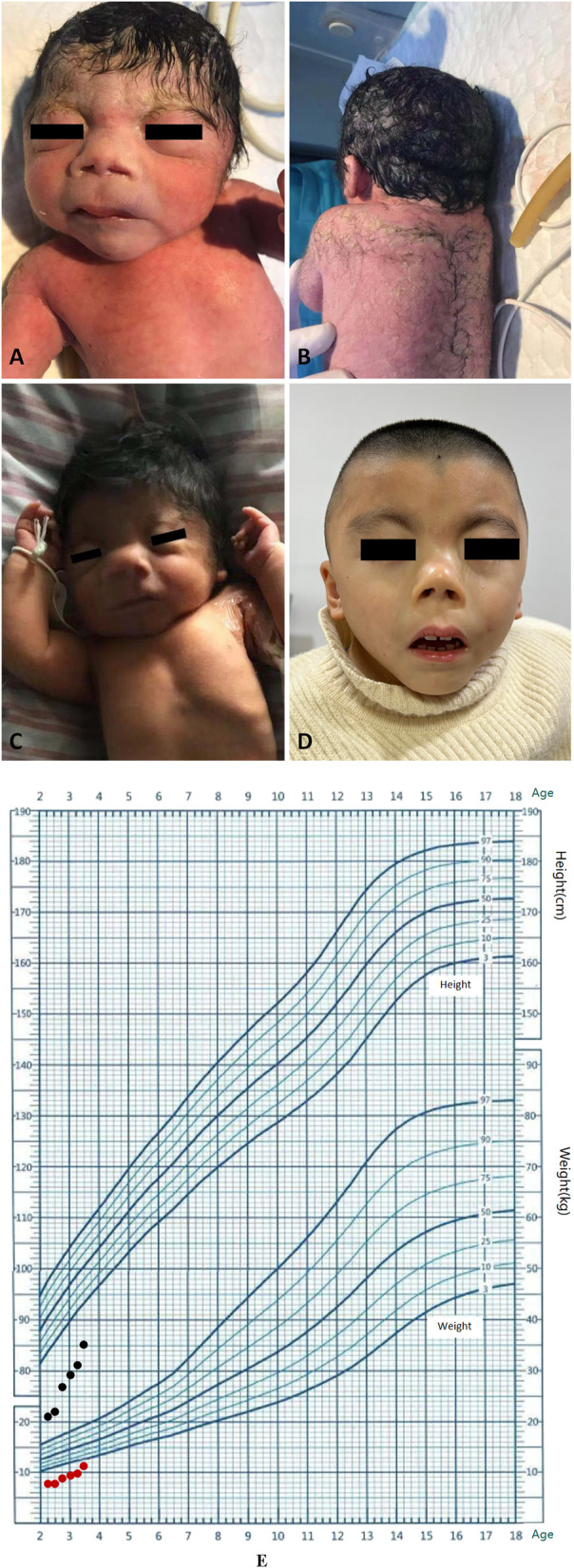
Clinical features of the proband. **(A)** Facial characteristics at birth. **(B)** Hypertrichosis at birth. **(C)** Facial features on 16th day of life. **(D)** Appearance at age of 3 years and 5 months. **(E)** Growth curve of children (from the age of 2 years and 2 months up to the age of 3 years and 5 months old, monitored every 3 months). The black dots represent changes in height. The red dots represent changes in weight.

The proband was delivered via cesarean section during placental abruption at 36^+^ weeks of gestation. The patient weighed 2050 g at birth (<10th percentile), had a body length of 40 cm (< third percentile), and with a head circumference indicating 30.8 cm, suggesting fetal growth restriction. The baby had dysmorphic facial features, including a low forehead hairline, a short and up-turn nose, an elongated philtrum, and a small lower jaw (Figure 1A). Other signs or symptoms also indicated a short neck, thickening of the occipital skin, shortened upper limbs, hairiness on the limbs and back, enorchia, and a micropenis (Figure 1B). Ultrasonography revealed an atrial septal defect (ASD), multiple cysts in both kidneys, separation of the left pelvis, and cryptorchidism. Cranial MRI showed an enlarged cisterna magna.

At follow-up, the patient exhibited distinct growth and intellectual disability. We also recorded typical facial characteristics at the age of 16th day of life and 3 years and 5 months of this child (Figures 1C,D). Spontaneous closure of the ASD was observed at 7 months of age. Surgery for cryptorchidism was performed at 2 years of age. He received recombinant human growth hormone (r-hGH) injection and regular rehabilitation training from the age of 2 years and 5 months old. Height and weight were monitored every 3 months Follow-up was performed up to 3 years and 5 months after the birth of this child, the height was only measured as 85 cm (< third percentile), in addition, the weight was merely 10 kg (< third percentile). The growth curve of this child is shown in Figure 1E. Motor assessments showed that the child could take a few steps alone, but the skills were unstable. He could grasp toy bricks and pass objects from one hand to the other. However, he could not put pellets into a bottle accurately. A deficiency in language development was also observed, and he could only unconsciously pronounce words. The Gesell Developmental Scale was used to comprehensively assess the development of the boy. When the proband at age of 3 years and 5 months old, developmental age of gross motorskills and adaptability is only 13 m, fine motor skills is 12.5 m, personal social activity is 13.4 m, and language developmental age is 10.3 m, suggesting a severe developmental delay.

### 3.2 Identification of NIPBL variant

**FIGURE 2:**
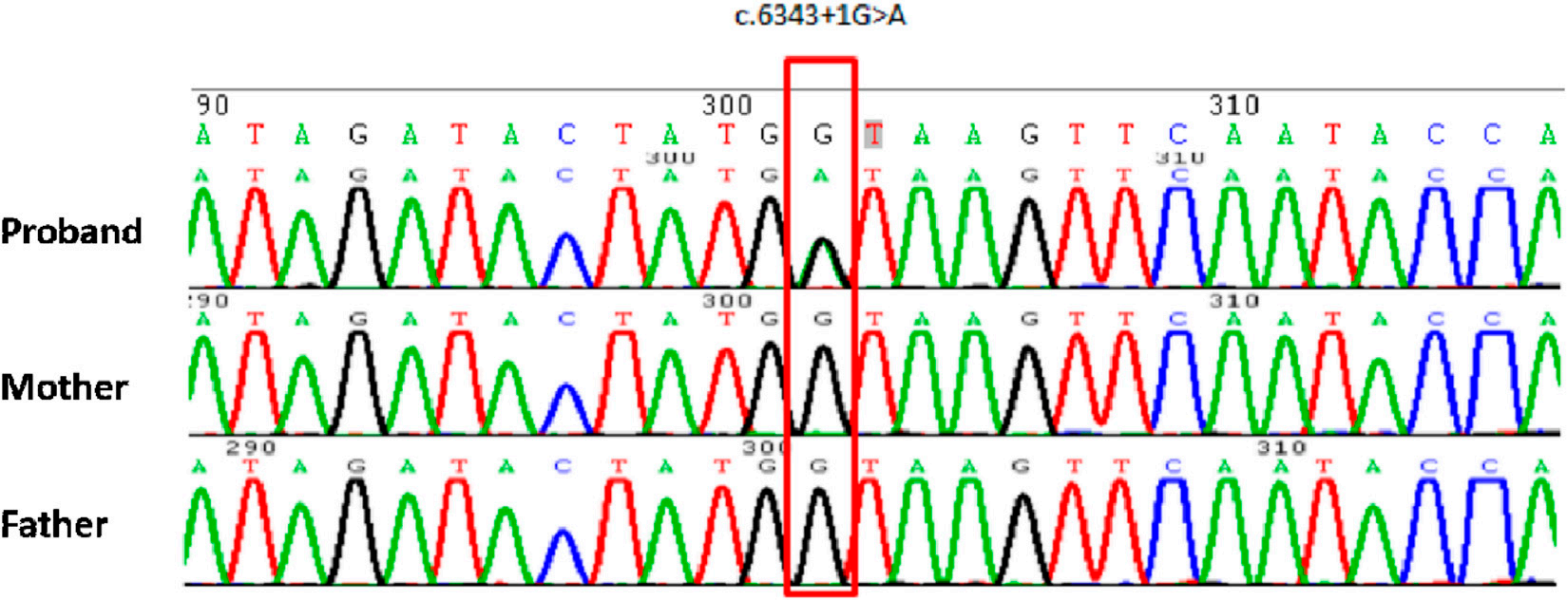
Sanger sequencing schematic diagram of the proband and his parents. The red frame indicates a variant position, which mutates into adenine(A) in proband, and in his parents are normal as guanine(G).

WES of the proband and his parents was performed to confirm the pathogenic genetic diagnosis in CdLS. The results revealed a novel splice-donor variant in intron 36 of the *NIPBL* gene (chr5:37044832, NM_133433.3) (Figure 2). Nucleobase guanine(G) changed to adenine(A) in the variant position of the proband. The variant was heterozygous in the proband, whereas the gene in his parents was wild-type, suggesting a *de novo* variant. The splice-donor variant could not ever be reported in the Human Exon Database (ExAC), Population Genome Mutation Frequency Database (gnomAD), 1,000 Genomes Database, or Human Gene Mutation Database (HGMD), indicating that it was novel and may be the cause of the disorder.

Analysis of the splice-donor variant suggested that the variant position is located in site +1 of intron36 in *NIPBL* (nucleobase guanine mutate into adenine), which could cause a distinct impact on mRNA splicing. Th splice-donor variant is regarded as a pathogenic variant that may impact the splicing process. We predicted the splicing disturbance and pathogenicity of this variant subsequently. The HSF and SpliceAI algorithms showed that the confidence score of the original donor site decreased in the splice-donor variant, suggesting that the variant may affect *NIPBL* splicing. The RDDCSC algorithm revealed that the variant may cause exon skipping, frameshift variant, and premature termination codon, indicating that it affected splicing in *NIPBL*.

### 3.3 RT-PCR analysis

**FIGURE 3:**
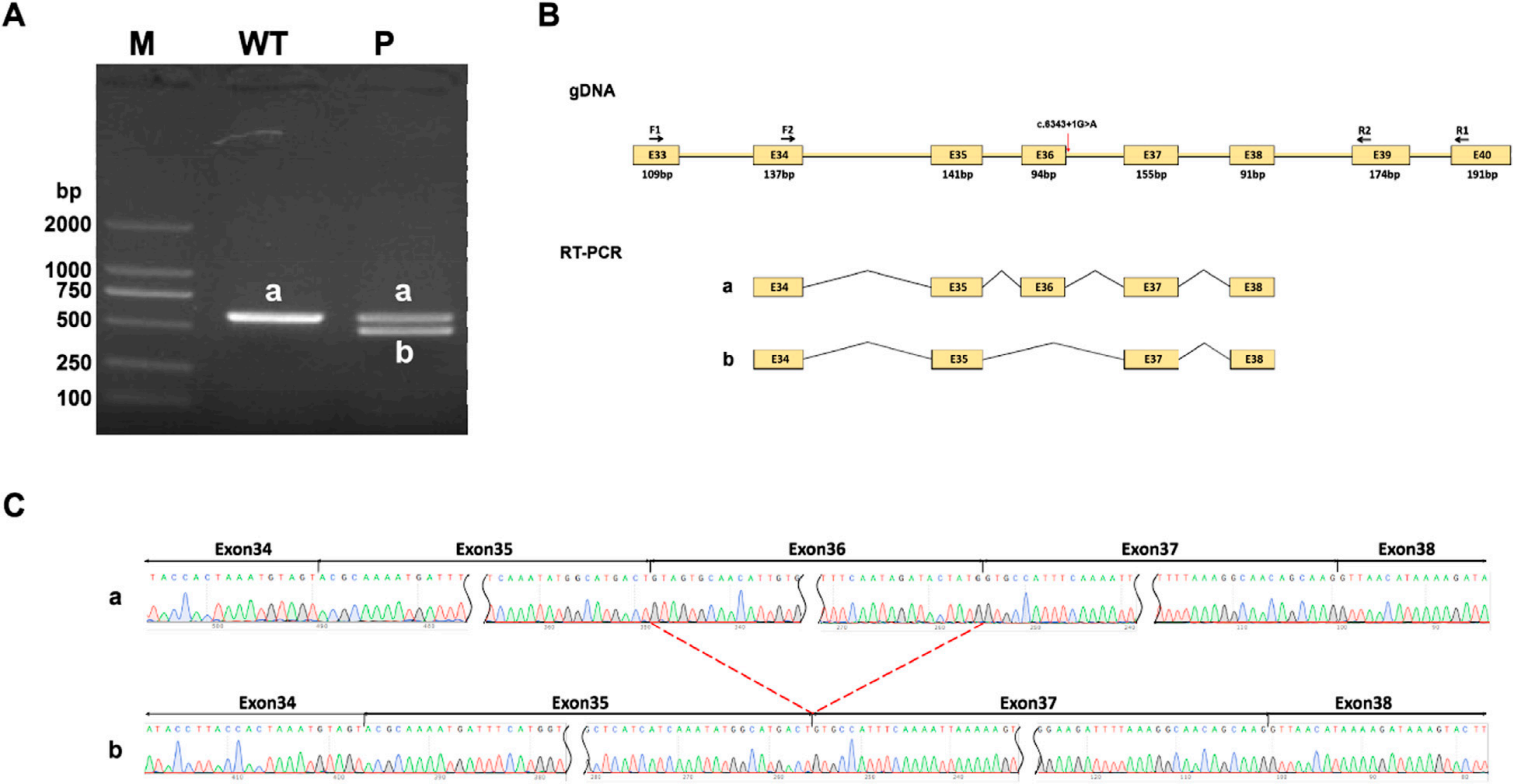
RT-PCR analysis. **(A)** RT-PCR products are visualized on gel electrophoresis. There exists only one longer band labeled “a” in his healthy mother (WT), while two bands labeled “a” and “b” are found in the proband (P). Bands “a” shows as normal size, Band “b” is shorter. **(B)** A schematic diagram of primer design and exon 36 skipping splicing, red arrow points variant position. **(C)** Sanger sequencing analysis of RT-PCR products. Band “b” is shorter than normal size band ‘a’ due to a loss of exon 36.

*NIPBL* is widely expressed in different tissues and whole blood samples. Bulk tissue gene expression analysis suggests the index of transcripts per million (TPM) is up to 7.818 in whole blood samples, which could be applied to RT-PCR for *in vitro* splicing analysis. In the control participant, amplified PCR products were visualized as a single longer band “a” on gel electrophoresis, by the normal band size. However, the proband had two different bands, “a” (which was the same as the normal one) and “b” (a shorter band), suggesting a heterozygous variant (Figure 3A). The shorter band was likely caused by exon 36 skipping due to the frameshift variant (Figure 3B). Sanger sequencing confirmed the effect of the variant on RNA splicing. The normal splicing form was presented as Exon34 (137bp)-Exon35 (141bp)-Exon36 (94bp)-Exon37 (155bp)-Exon38 (91bp) in the control, whereas abnormal splicing was observed as Exon34 (137bp)-Exon35 (141bp)-Exon37 (155bp)-Exon38 (91bp) in the variant, confirming the loss of exon 36 (Figure 3C).

### 3.4 Minigene splicing assay results

**FIGURE 4:**
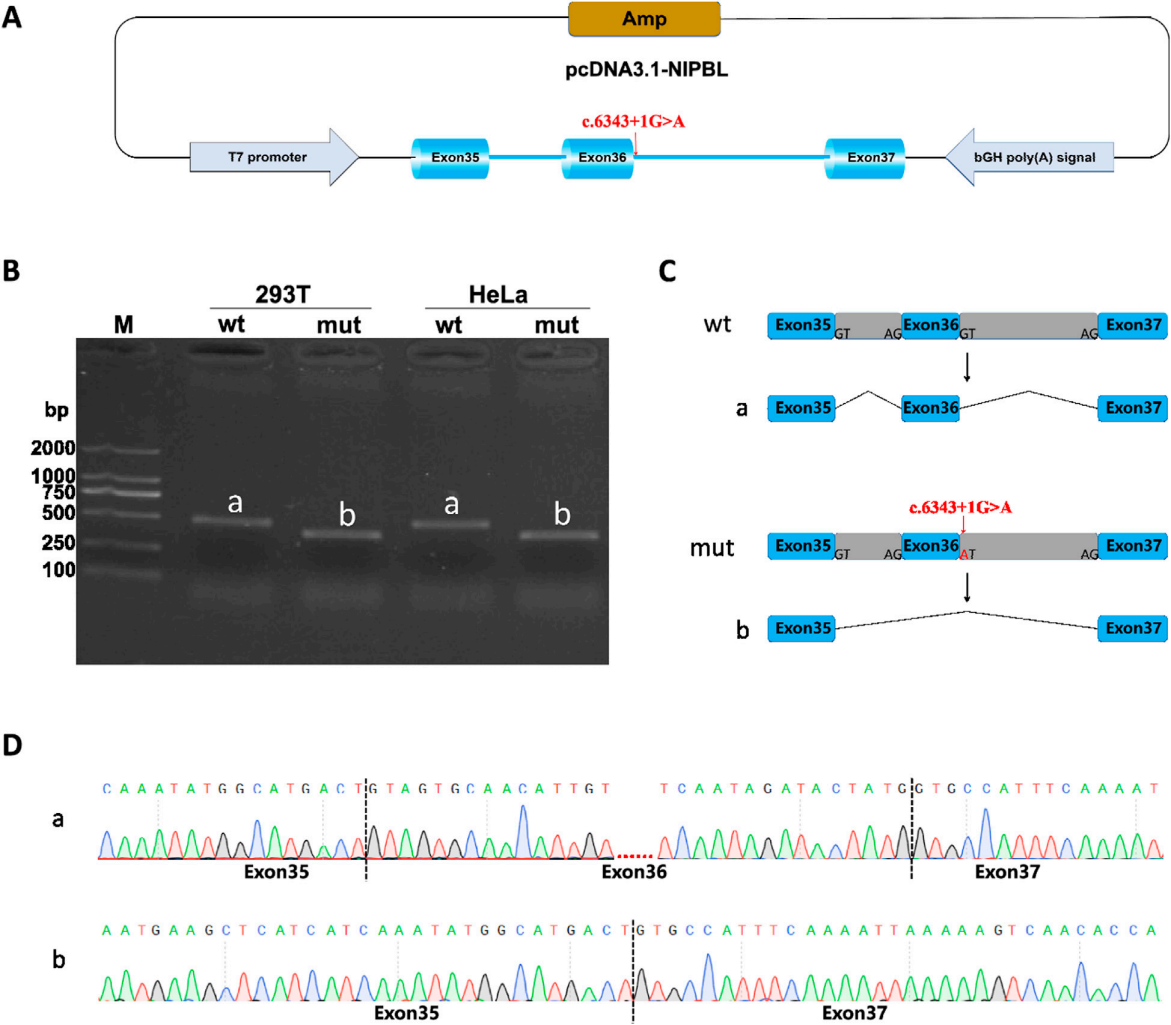
Minigene splicing assay analysis of *NIPBL*-pcDNA3.1. **(A)** A schematic diagram of *NIPBL*-pcDNA3.1 construction. **(B)** Minigene products of *NIPBL*-pcDNA3.1 are separated through gel electrophoresis. The longer bands are labeled “a” (wt) and shorted bands are labeled “b” (mut) in HeLa and 293T cells. Bands “a” are normal size, Bands “b” are shorter. **(C)** Schematic diagram of minigene splicing in *NIPBL*-pcDNA3.1. **(D)** Sanger sequencing analysis of minigene products. Bands ‘b’ are shorter than normal size bands ‘a’ due to a deletion of exon 36.

**FIGURE 5:**
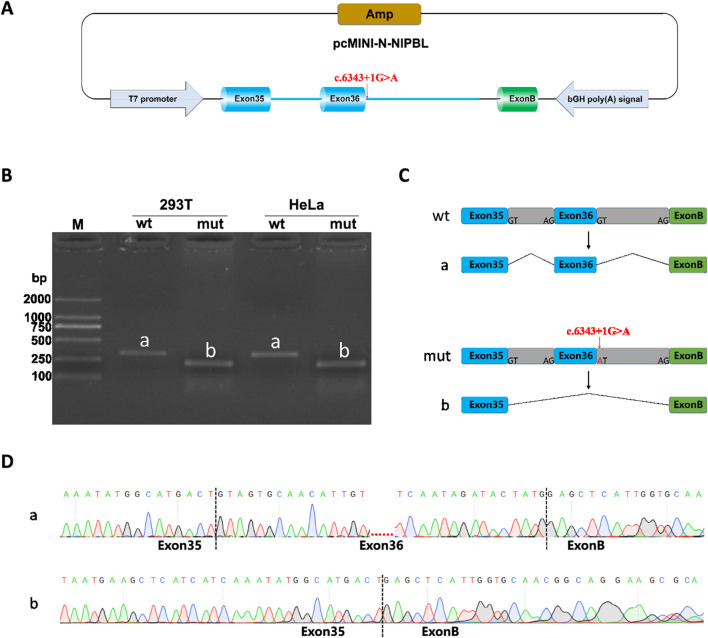
Minigene splicing assay analysis of *NIPBL*-pcMINI-N. **(A)** A schematic diagram of *NIPBL*-pcMINI-N construction. **(B)** Minigene products of *NIPBL*-pcMINI-N are separated through gel electrophoresis. The longer bands are labeled “a” (wt) and shorted bands are labeled “b” (mut) in HeLa and 293T cells. Bands “a” are normal size, Bands “b” are shorter. **(C)** Schematic diagram of minigene splicing in *NIPBL*-pcMINI-N. **(D)** Sanger sequencing analysis of minigene products. Bands “b” are shorter than normal size bands “a” due to a deletion of exon 36.

Minigene splicing assay (*in vitro*) was also performed to further verify the function of the splice-donor variant on the splicing process. *NIPBL*-pcDNA3.1-wt/mut and *NIPBL*-pcMINI-N-wt/mut recombinants were successfully constructed as shown in diagrams (Figure 4A; Figure 5A). Gel electrophoresis showed a larger normal band named “a” in the wild type group, meanwhile, a smaller single band defined as “b” in the variant. In *NIPBL*-pcDNA3.1-wt/mut constructions, a single 465 bp band was observed in the wild-type, in line with the normal size as expected, whereas a shorter band “b” was produced in the variant (Figure 4B). Similarly, in the constructions of *NIPBL*-pcMINI-N-wt/mut, the normal band “a” was 315 bp size in the wild-type, revealing normal mRNA splicing, while a smaller band “b” was produced with abnormal splicing in the variant (Figure 5B). Therefore, the splice-donor variant may cause exon 36 skipping (Figures 4C, 5C). Sequencing results confirmed that the splice-donor variant caused the deletion of exon 36 in *NIPBL*. Sequencing analysis revealed disturbed RNA splicing of Exon35 (141bp)-Exon37 (155bp) in *NIPBL*-pcDNA3.1-mut (Figure 4D). Similarly, the abnormal splicing caused by the variant in the *NIPBL*-pcMINI-N-mut constructs was presented as Exon35 (141bp)-ExonB (57bp) (Figure 5D). These results indicate that the splice-donor variant causes the loss of exon 36 and affects normal mRNA splicing.

## 4 Discussion

Many studies have shown that abnormal expression of *NIPBL* leads to abnormal developmental in the heart, limbs, and nervous system (Garfinkle and Gruber, 2019; Mazzola et al., 2019). Because *NIPBL* interferes with the function of *MAU2* as well as the expression of *HOX*, participating in craniofacial development and thus resulting in limb growth, *NIPBL* variants can cause limb malformations (Muto et al., 2014; Smith et al., 2014). Pathogenic variants in the *NIPBL* gene lead, to varying degrees, to a reduction in normally functional *NIPBL* or even haploinsufficiency. The alteration of the *NIPBL* gene leads to the severe clinical features of CdLS, the so-called “classical phenotype. Individuals with pathogenic *NIPBL* variants always show a classical and more severe in CdLS phenotypic (Krantz et al., 2004). Patients diagnosed with CdLS have been reported to have *de novo* heterozygous pathogenic variants in most cases, but are not inherited from their parents. CdLS variants result in perturbation of gene expression and thus interfere with global transcription (Sarogni et al., 2020; Yuan et al., 2015).

Missense or nonsense variants, splicing changes, small deletions, and insertions in the *NIPBL* variants account for approximately 95% of the *NIPBL* variant reports (HGMD database). Point variants or single-nucleotide variants in *NIPBL* account for the majority and large-scale genomic rearrangements are rarely reported (Bhuiyan et al., 2007; Ratajska et al., 2010). The clinical severity of CdLS depends on the dosage effect of the gene, which is crucial to influencing the clinical presentation. Truncation, nonsense, splice site, and frameshifts in *NIPBL* variants contribute to a truncated and possibly non-functional *NIPBL* protein pathogenic variant associated with a severe CdLS clinical phenotype; however, missense variants generally result in a milder CdLS pattern, and individuals with large deletions associated with CdLS show more severe clinical symptoms; this grading indicates that *NIPBL* is sensitive to gene dosage variants (Selicorni et al., 2021).

This study investigated a child who was diagnosed with CdLS at birth. WES identified a new *NIPBL* variant site (c.6343 + 1G > A), but this was not observed in the general population. This was a heterozygous *de novo* variant that could not be found among his parents. This variant is located in the +1 site of intron 36 and is known as a splicing impact. In our study, RT-PCR and minigene splicing experiments were performed to verify the effect of the splice-donor variant on mRNA splicing. This variant affects the normal splicing of the mRNA of the *NIPBL* gene, resulting in exon 36 skipping and frameshift variants, creating a premature stop codon (PTC). PTC may lead to non-sense-mediated mRNA degradation or the production of truncated proteins. In this study, a truncated protein with a length of 2088 amino acids (protein with a normal length of 2,804 aa) was produced (c. 6250_6343 delp. Val2085Profs*5). NIPBL is a large protein of more than 2,500 amino acid residues, comprises unstructured regions and several HEAT repeats. The large genomic size and complex domain architectures of NIPBL pose significant challenges for protein-level validation. In our study, the variant results in a substitution of valine to proline at position 2085, occurs in a structurally critical motif. Our next steps will prioritize protein-level validation to elucidate the impact of this variant. This patient had a relatively typical clinical phenotype consistent with the *NIPBL* genotype: dysmorphic facial features, short neck, occipital skin thickening, upper limb deformities, polytheism, cryptorchidism and micropenis; in addition, the more severe manifestations of the patient may be closely related to nonsense variants caused by the *NIPBL* gene variant, as was previously described, various gene dosage variants in the gene usually differ in the severity of the CdLS phenotype.

Growth retardation, short stature, and delayed puberty are also relatively common symptoms in children with CdLS (Kline et al., 1993; Kline et al., 2007), recently, a girl with a *de novo* splicing variant in the *NIPBL* gene was treated with r-hGH at age 4.3 years. Treatment with r-hGH resulted in a height increase of 1.6 SD score (de Graaf et al., 2017), suggesting that hormonal therapy may be effective in CdLS patients with short stature. In this case, the child was born 40 cm (<third percentile) and the child received r-hGH injection at the age of 2 years and 5 months. From follow-up until 3 years and 5 months after the birth of the child, the height was still only 86 cm (<the third percentile), indicating the effect of growth hormone therapy in this child. Longitudinal growth monitoring demonstrated significant height improvement from 73 cm (−4.5 SD) at 2 years 5 months–86 cm (−2.9 SD) at 3 years 5 months, representing a 13 cm gain over this 1-year treatment period. Although the patient’s height remained below the third percentile, this substantial catch-up growth suggests potential therapeutic efficacy of the growth hormone intervention. Insulin-like Growth Factor 1(IGF-1) levels increased significantly from 96 ng/mL to 228 ng/mL during treatment, demonstrating biochemical responsiveness to growth hormone therapy. In the present case, the short duration of rhGH therapy (1 year) represents a notable limitation, as this timeframe may be insufficient to fully demonstrate the treatment’s growth-promoting effects in severe growth retardation. Previous longitudinal studies have demonstrated that extended growth hormone treatment courses (e.g., 8-year follow-up periods in de Graaf et al. (2017)) are required to fully evaluate therapeutic efficacy, particularly for demonstrating sustained improvements in final adult height. The initial recombinant growth hormone dose in our proband (0.73 mg/m^2^/day) was 15% lower than the reference standard of 0.86 mg/m^2^/day established in the positive case in de Graaf et al. (2017). Children with CdLS may demonstrate reduced sensitivity to rhGH therapy, potentially necessitating higher-than-standard dosing regimens to achieve optimal growth outcomes. Based on these findings, dose escalation will be considered for future interventions to optimize growth outcomes. A survey on CdLS showed that most of the children had feeding problems (Sarimski, 1997); combined with the weight gain of 2050 g at birth (<10th percentile), and only 10 kg (<third percentile), due to poor feeding practices, multisystem diseases, and surgical trauma (cryptorchidism). Feeding difficulties and reduced basal metabolic rate may synergistically exacerbate growth retardation in this patient. Moreover, the splice-donor variant in this case is previously unreported, so it remains unknown whether children with variants at this site are sensitive to growth hormone therapy. Mild phenotype characterized by isolated growth retardation with preserved intellectual function in 2017 reported CdLS case, while severe phenotypic manifestation featuring global developmental delay (e.g., growth restriction, cognitive impairment, and facial dysmorphism). The milder case caused an in-frame deletion which typically retains partial protein function, while nonsense and frameshift mutations may cause a sever phenotype. Our proband carries a frameshift variantion, predicted to cause a truncated protein. This genotype-phenotype gradient correlates with observed GH therapy outcomes, better response in mild genotype, attenuated response in severe genotype. This genotypic gradient (mild → severe) parallels both phenotypic severity and GH response, supporting a unified model where variation class dictates clinical trajectory. Precision dosing strategies, informed by genetic profiling, may be warranted for severe genotypes.

Classical children with CdLS also show developmental delays, including motor development, as well as intellectual development. This case was evaluated at the age of 3 years and 5 months of age. The results showed that the developmental quotient of the children was only 31 points, and the developmental age of adaptability, large motor, fine motor, language, and personal-social aspects was only equivalent to the level of 10–13 months of age. This suggests that the child had significant developmental disorders in movement, language, and social interaction. A survey reported that only four out of the 27 children with CdLS were able to communicate using language, but most of the older children expressed their needs (Sarimski, 1997) in non-verbal means. Studies have reported that most children with CdLS have mild to severe intellectual disabilities as well as autistic features (Srivastava et al., 2014; Basile et al., 2007). CdLS is characterized by autistic traits, particularly excessive repetitive behavior, and expressive language deficits. Communication and social deficits are exacerbated with age compared to their neurotypical peers. The results of the current Gesell development scale of the children in this case suggest severe defects. Therefore, in the future follow-up process, attention should be paid to their communication skills and social skills, to early detect whether the children have autism tendencies and intervene in advance.

## 5 Conclusion

In conclusion, we identified a novel splice-donor variant (*NIPBL* c.6343+1G > A) that causes a typical CdLS, and revealed its effect on RNA splicing. Providing strong evidence for a definitive diagnosis of this patient. The findings of this study further expand the spectrum of pathogenic variant in CdLS. This study provides insight into genotype–phenotype correlations. Furthermore, the results of this study may lay the foundation for later mechanism research and contribute to future therapeutic approaches of CdLS.

## 6 Limitation

Currently, there is only one case, demonstrates the correlation between this novel intronic variant of *NIPBL* gene and Cornelia de Lange syndrome. It is difficult to identify the accurate relationship of genotype and phenotype in absence of more case reports. The functional interpretation of these splicing variants is presently limited to the RNA level, pending subsequent protein characterization studies.

## Data Availability

The datasets presented in this study can be found in online repositories. The names of the repository/repositories and accession number(s) can be found below: https://www.ncbi.nlm.nih.gov/, SRA accession:SRR31906592.
