# Supplementary material for: Case Report: A novel intronic variant of NIPBL gene detected in a child with cornelia de lange syndrome
Source: Front Genet. 2025 Sep 10;16:1665167. doi: 10.3389/fgene.2025.1665167 (PMC12457154; doi:10.3389/fgene.2025.1665167)
Supplement: Supplementary file 1 [file DataSheet1.pdf]

CARE Checklist

1.**Title**：Case report：A novel intronic variant of *NIPBL* gene detected in a child with Cornelia de Lange syndrome

2.**Key Words**：Cornelia de Lange syndrome, variant, *NIPBL*, splicing

3.**Abstract**：**Background:** Cornelia de Lange syndrome (CdLS) is a genetically heterogeneous disorder involving multi-system organs, causing physical and mental congenital malformation. Nipped-B-like protein (*NIPBL*) variants are associated with various CdLS phenotypes. Newborns with typical clinical manifestations (developmental retardation, special appearances, and limb malformation) require a diagnosis. However, diagnosing CdLS is challenging on account of its heterogeneity of genotype and phenotype. **Methods:** In this study, molecular analysis was applied, containing whole exome sequencing (WES), reverse transcriptase PCR (RT-PCR), and minigene splicing assays. **Results:**WES revealed a novel heterozygous variant (chr5:37044832, NM_133433.3,c.6343+1G>A) on intron 36 of *NIPBL*. RT-PCR and minigene splicing assays were performed to identify the function of the mutation of c.6343+1G>A on subsequent RNA splicing. The mutation caused exon 36 to be skipped. A premature termination codon (PTC) appeared subsequently and a truncated protein with a length of 2088 aa was produced. **Conclusion:** a novel pathogenic variant of CdLS is identified, which affects normal mRNA splicing of the *NIPBL* gene. These findings enrich the knowledge of CdLS gene mutations, which may be responsible for developing this rare disease.

4.*Introduction*：Cornelia de Lange syndrome (CdLS), a rare congenital disease of the multisystem caused by genetic mutations, has an extremely low incidence of one estimated CdLS per 10,000 to 30,000 live births. Children with CdLS have typical clinical manifestations such as facial features, hypertrichosis, growth restriction, intellectual disability, and upper limb deformity . Occipital skin thickening was found in nearly 51% of patients, with special appearance features accounting for nearly half of . Approximately 30% of the patients were reported to have an upper limb deficit. Although cardiac abnormalities are not the primary standard and typical clinical manifestation of the disease, approximately 50% of patients with CdLS present with congenital heart defects . An international consensus statement created a scoring system defining CdLS as both classical and non-classical CdLS. Classical CdLS, due to unique facial features, growth limitations, and limb deformities, can be identified at birth by experienced pediatricians or clinical geneticists. However, due to the heterogeneous genotype and phenotype of CdLS, some non-classical CdLS do not have characteristic clinical manifestations, thus bringing great difficulties for clinical diagnosis.

As currently reported in the literature, CdLS is due to mutations in the following genes, *NIPBL, SMC1A, SMC3, RAD21, HDAC8, BRD4,* and *ANKRD11*. *SMC1A* mutations are found in about 5% of patients with CdLS and the mutations in *HDAC8, RAD21,* and *SMC3* totaled about 5%; *BRD4* and *ANKRD11* gene mutations are newly discovered in recent years, being too small to count the percentage; The clinical manifestations of several mutations are less typical . Mutations in the *NIPBL* gene are detected in 60% of patients with CdLS . The *NIPBL* gene is located on 5p13.2 and encodes for a triangular protein that plays an important role in the chromatid cohesion process and enhancer-promoter communication. Potential pathogenic variants in the *NIPBL* gene have been tightly associated with the typical manifestation of CdLS.

This study reports a classic case of CdLS diagnosed from birth in a child who also had a mutation

in the *NIPBL* gene, but we detected a novel intronic mutation (chr5:37044832, NM_133433.3,c.6343+1G> A).

5.**Patient Information**： The proband was a newborn, male, The proband was delivered via cesarean section during placental abruption at 36^{+} weeks of gestation.

6.**Clinical Findings**: The patient weighed 2050 g at birth (< 10th percentile), had a body length of 40 cm (< third percentile), and with a head circumference indicating 30.8 cm, suggesting fetal growth restriction. The baby had dysmorphic facial features, including a low forehead hairline, a short and up-turn nose, an elongated philtrum, and a small lower jaw . Other signs or symptoms also indicated a short neck, thickening of the occipital skin, shortened upper limbs, hairiness on the limbs and back, enorchia, and a micropenis .

7.**Timeline**

Date	Event
2019.8.14	The newborn proband had a fetal growth restriction and had dysmorphic facial features
2019.8.17	Cranial MRI showed an enlarged cisterna magna
2019.8.18	Ultrasonography revealed an atrial septal defect (ASD)
2019.8.19	Ultrasonography revealed cryptorchidism
2019.9.17	Whole exome sequencing (WES) revealed a novel variant, c.6343+1G>A, in intron 36 of the *NIPBL* gene
2020.4.8	Spontaneous closure of the ASD
2021.11.8	Surgery for cryptorchidism
2022.1.12	received recombinant human growth hormone injection and regular rehabilitation training
2023.2.11	Last follow-up：Photo record, measurement, Gesell Developmental Scale
2024.4.30	Splicing analysis of this novel *NIPBL* variant

8.**Diagnostic Assessment**: Ultrasonography revealed an atrial septal defect (ASD), multiple cysts in both kidneys, separation of the left pelvis, and cryptorchidism. Cranial MRI showed an enlarged cisterna magna. Whole exome sequencing of the proband and his parents was performed to confirm the pathogenic genetic diagnosis in CdLS. The results revealed a novel variant, c.6343+1G>A, in intron 36 of the *NIPBL* gene (NM_133433.4) .Nucleobase guanine(G) changed to adenine(A) in the mutation site of the proband. The mutation was heterozygous in the proband, whereas the gene in his parents was wild-type, suggesting a de novo mutation. This variant (c.6343+1G>A) could not ever be reported in the Human Exon Database (ExAC), Population Genome Mutation Frequency Database (gnomAD), 1000 Genomes Database, or Human Gene Mutation Database (HGMD), indicating that it was novel and may be the cause of the disorder. Analysis of the variant (c.6343+1G>A) suggested that the mutation site is located in site +1 of intron36 in *NIPBL* (nucleobase guanine mutate into adenine), which could cause a distinct impact on mRNA splicing. This novel *NIPBL* variant is regarded as a pathogenic mutation that may impact the splicing process. We predicted the splicing disturbance and pathogenicity of

this mutation subsequently. The HSF and SpliceAI algorithms showed that the confidence score of the original donor site decreased after mutation, suggesting that the mutation may affect NIPBL splicing. The RDDCSC algorithm revealed that the mutation may cause exon skipping, frameshift mutations, and premature termination codon, indicating that it affected splicing in *NIPBL*.

9. **Therapeutic Intervention**: Surgery for cryptorchidism was performed at two years of age.He received recombinant human growth hormone injection and regular rehabilitation training from the age of 2 years and 5 months old.

10.**Follow-up and Outcomes**: Spontaneous closure of the ASD was observed at 7 months of age. Surgery for cryptorchidism was performed at two years of age.He received recombinant human growth hormone injection and regular rehabilitation training from the age of 2 years and 5 months old. Height and weight were monitored every 3 months Follow-up was performed up to 3 years and 5 months after the birth of this child, the height was only measured as 85 cm (< third percentile), in addition, the weight was merely 10 kg (< third percentile). The growth curve of this child is recorded. The Gesell Developmental Scale was used to comprehensively assess the development of the boy, suggesting a severe developmental delay.

11.**Discussion**: Many studies have shown that abnormal expression of *NIPBL* leads to abnormal developmental in the heart, limbs, and nervous system. Because *NIPBL* interferes with the function of MAU2 as well as the expression of HOX, participating in craniofacial development and thus resulting in limb growth, NIPBL mutations can cause limb malformations. Pathogenic mutations in the *NIPBL* gene lead, to varying degrees, to a reduction in normally functional *NIPBL* or even haploinsufficiency. The alteration of the *NIPBL* gene leads to the severe clinical features of CdLS, the so-called "classical phenotype. Individuals with pathogenic *NIPBL* variants always show a classical and more severe in CdLS phenotypic. Patients diagnosed with CdLS have been reported to have de novo heterozygous pathogenic mutations in most cases, but are not inherited from their parents. CdLS mutations result in perturbation of gene expression and thus interfere with global transcription.

Missense or nonsense mutations, splicing changes, small deletions, and insertions in the *NIPBL* mutations account for approximately 95% of the *NIPBL* mutation reports (HGMD database). Point mutations or single-nucleotide variants in *NIPBL* account for the majority and large-scale genomic rearrangements are rarely reported. The clinical severity of CdLS depends on the dosage effect of the gene, which is crucial to influencing the clinical presentation. Truncation, nonsense, splice site, and frameshifts in *NIPBL* mutations contribute to a truncated and possibly non-functional *NIPBL* protein pathogenic variant associated with a severe CdLS clinical phenotype; however, missense mutations generally result in a milder CdLS pattern, and individuals with large deletions associated with CdLS show more severe clinical symptoms; this grading indicates that *NIPBL* is sensitive to gene dosage mutations.

This study investigated a child who was diagnosed with CdLS at birth. WES identified a new *NIPBL* variant site (c.6343 + 1G> A), but this was not observed in the general population. This was a heterozygous de novo mutation that could not be found among his parents. This mutation is located in the + 1 site of intron 36 and is known as a splicing impact. In our study, RT-PCR and minigene splicing experiments were performed to verify the effect of the mutation (c.6343 + 1G> A) on mRNA splicing. This mutation affects the normal splicing of the mRNA of the *NIPBL* gene, resulting in exon 36 skipping and frameshift mutations, creating a premature stop codon (PTC).

PTC may lead to non-sense-mediated mRNA degradation or the production of truncated proteins. In this study, a truncated protein with a length of 2088 amino acids (protein with a normal length of 2804 aa) was produced (c. 6250_6343 delp.Val2085Profs*5). This patient had a relatively typical clinical phenotype consistent with the *NIPBL* genotype: dysmorphic facial features, short neck, occipital skin thickening, upper limb deformities, polytheism, cryptorchidism and micropenis; in addition, the more severe manifestations of the patient may be closely related to nonsense mutations caused by the *NIPBL* gene variant (c.6343 + 1G> A), as was previously described, various gene dosage mutations in the gene usually differ in the severity of the CdLS phenotype.

Growth retardation, short stature, and delayed puberty are also relatively common symptoms in children with CdLS; recently, a girl with a de novo splicing mutation in the *NIPBL* gene was treated with recombinant human growth hormone (r-hGH) at age 4.3. Treatment with r-hGH resulted in a height increase of 1.6 SD score, suggesting that hormonal therapy may be effective in CdLS patients with short stature. In this case, the child was born 40 cm (<3rd percentile) and the child received recombinant human growth hormone injection at the age of 2 years and 5 months. From follow-up until 3 years and 5 months after the birth of the child, the height was still only 85 cm (<the third percentile), indicating the effect of growth hormone therapy in this child. A survey on CdLS showed that most of the children had feeding problems; combined with the weight gain of 2050 g at birth (<10th percentile), and only 10 kg (<3rd percentile), due to poor feeding practices, multisystem diseases, and surgical trauma (cryptorchidism). Moreover, the gene mutation site in this case is a new mutation, so it remains unknown whether children with mutations at this site are sensitive to growth hormone therapy.

Classical children with CdLS also show developmental delays, including motor development, as well as intellectual development. This case was evaluated at the age of 3 years and 5 months of age. The results showed that the developmental quotient of the children was only 31 points, and the developmental age of adaptability, large motor, fine motor, language, and personal-social aspects was only equivalent to the level of 10-13 months of age. This suggests that the child had significant developmental disorders in movement, language, and social interaction. A survey reported that only four out of the 27 children with CdLS were able to communicate using language, but most of the older children expressed their needs in non-verbal means. Studies have reported that most children with CdLS have mild to severe intellectual disabilities as well as autistic features. CdLS is characterized by autistic traits, particularly excessive repetitive behavior, and expressive language deficits. Communication and social deficits are exacerbated with age compared to their neurotypical peers. The results of the current Gesell development scale of the children in this case suggest severe defects. Therefore, in the future follow-up process, attention should be paid to their communication skills and social skills, to early detect whether the children have autism tendencies and intervene in advance.

Limitation: Currently, there is only one case, demonstrates the correlation between this novel intronic variant of *NIPBL* gene and Cornelia de Lange syndrome. It is difficult to identify the accurate relationship of genotype and phenotype in absence of more case reports.

12.**Patient Perspective**: The family expressed expressing acceptance after obtaining a genetic diagnosis and positively received recombinant human growth hormone injection treatment.

13. **Informed Consent**: Informed consent was obtained from all subjects involved in the study.
